# A fetal fraction enrichment method reduces false negatives and increases test success rate of fetal chromosome aneuploidy detection in early pregnancy loss

**DOI:** 10.1186/s12967-022-03555-9

**Published:** 2022-08-02

**Authors:** Longwei Qiao, Bin Zhang, Xiaojuan Wu, Chunhua Zhang, Ying Xue, Hui Tang, Haoyu Tang, Jingye Shi, Yuting Liang, Bin Yu, Ting Wang

**Affiliations:** 1 Center for Reproduction and Genetics, School of Gusu, The Affiliated Suzhou Hospital of Nanjing Medical University, Suzhou Municipal Hospital, Nanjing Medical University, Suzhou, Jiangsu Province China; 2 Changzhou Maternal and Child Health Care Hospital, Changzhou Medical Center, Nanjing Medical University, No. 16 Dingxiang road, Changzhou, Jiangsu Province China; 3 Center for Clinical Laboratory, The First Affiliated Hospital of Soochow University, Suzhou, Jiangsu Province China

**Keywords:** Cell-free DNA, Fetal chromosomal aneuploidy, Fetal fraction enrichment method, Early pregnancy loss

## Abstract

**Objective:**

We and others have previously demonstrated that the size-selection enrichment method could remarkably improve fetal fraction (FF) in the early gestational age (GA, 12–13 weeks), suggesting that 9 or 10 weeks should not be used as a threshold for GA in size-selection noninvasive prenatal screening (NIPS). Here, we assessed whether this method was reliable for detecting fetal chromosomal aneuploidy at the earliest GA (6–8 weeks).

**Methods:**

Size-selection NIPS for fetal chromosomal aneuploidy was applied to 208 pregnancy plasma samples (102 male and 106 female fetuses), while the 169 pregnancy samples with male fetuses also underwent standard NIPS. Multivariable linear regression models were used to evaluate the association between fold-change of FF and experimental factors.

**Results:**

The sensitivity of the cell-free DNA (cfDNA) test in detecting aneuploidy was 100% when screened with FF enrichment, whereas the sensitivity of the same patients was only 62.5% (5/8) without FF enrichment. In the 102 pregnancy samples with male fetuses, FF increased from 6.1% to 15.7%, and the median increase in FF was 2.8-fold with enrichment. Moreover, there was a trend toward an increasing success rate of the cfDNA test from 6 to 13 weeks of gestation, especially when the test success rate reached 100% after 7 weeks with FF enrichment. Multivariate linear regression analysis demonstrated that a lower initial FF, shorter cfDNA size, increased body mass index (BMI), and later GA were all independent predictors of a higher fold-change of FF. Compared with ≤ 120 bp cfDNA fragments, the mean fold-change of FF differences was 0.820 for 121–125 bp, 0.229 for 126–130 bp, − 0.154 for 131–135 bp, − 0.525 for 136–140 bp and − 0.934 for > 140 bp (*P*_*trend*_ < 0.0001), suggesting that fold-change of FF significantly decreased with cfDNA fragments > 125 bp. These results were statistically significant after adjusting for confounding factors in the models for fold-change of FF.

**Conclusions:**

The FF enrichment method is a reasonable strategy to detect fetal chromosomal aneuploidy in early pregnancy loss with reduced false negatives and increased test success rate after 7 weeks of GA and should be recommended for patients with early pregnancy loss.

**Supplementary Information:**

The online version contains supplementary material available at 10.1186/s12967-022-03555-9.

## Introduction

Miscarriage, defined as the loss of pregnancy naturally before 20 weeks of gestation, usually occurs during the first trimester and in approximately 15% of clinically recognized pregnancies [1]. Approximately 50% of early pregnancy losses are caused by chromosome abnormalities that can be detected by using conventional karyotyping or chromosomal microarray (CMA). Trisomies are the most commonly detected chromosomal abnormalities (59.7%), followed by polyploidies (22%), monosomies (7.5%), unbalanced structural abnormalities (7%), and multiple aneuploidies (3.8%) [2]. However, the success rate of products of conception karyotyping may be as low as 53% because of the high rate of culture failure (32%) [3] and maternal contamination (15%) [4]. Other limitations include the extraction of nonviable tissues. It has therefore been suggested that the use of CMA based on pregnancy tissue DNA analysis improves test success rates and provides informative results in 95% of cases; however, CMA is expensive. Moreover, the quality of the products of conception will impact the success rate of both the conventional karyotyping and CMA [1].

Recently, Yaron et al*.* [5] proposed an interesting strategy based on prenatal cell-free DNA (cfDNA) screening, which could serve as an alternative to products of conception genetic analysis to guide further management of patients with early pregnancy losses. The authors evaluated 86 patients experiencing early pregnancy loss with complete cytogenetic results in products of conception and available genome-wide cfDNA testing results. Of these, a chromosomal anomaly was detected in 55 patients (64%). cfDNA testing had a sensitivity and specificity of 82% (45/55) and 90% (28/31), respectively [5–7]. They also performed cost-effective analyses of different testing pathways for early pregnancy loss, which demonstrated that in comparison with existing cytogenetic testing on products of conception, the cfDNA analysis pathway allowed for better sample accessibility at a lower cost per patient [8]. However, even after chromosome-specific standard log likelihood ratio (LLR) threshold corrections, cfDNA testing is unable to detect 18% of aneuploidies; the major reason for this false negative is low fetal fraction (FF).

Previous studies have reported that fetal-derived DNA fragments tend to be shorter than the maternal-derived ones, which suggests that leveraging the size difference at the bioinformatics and molecular levels has the potential to enrich the FF of the sample [9]. On this basis, we and others developed an effective noninvasive prenatal screening (NIPS) method to preferentially sequence shorter cfDNA fragments to significantly enrich the FF, which shows an increased success rate and a reduced false negative rate compared to the standard NIPS method [10–12]. Specifically, Hu et al. described cfDNA enrichment was performed before adaptor ligation during library construction [11]. After enrichment, the FF increased to 22.6 ± 6.6%. Welker et al. and our group developed a new cfDNA enrichment that size selection for the cfDNA library fragment < 140 bp by E-Gel^®^ Agarose Gel Electrophoresis System to notably improve the FF, which increased to 30.7%, suggesting that the enrichment effect of this method may be better [10, 12]. Therefore, in this study, we aimed to investigate whether this enrichment method is reliable for detecting fetal chromosomal aneuploidy during the earliest gestational age (GA, 6–8 weeks).

## Materials and methods

### Study population

Plasma samples from 208 pregnant women were collected at two prenatal diagnostic centers between August 2019 and December 2021. Plasma samples of pregnant women with male fetuses or early pregnancy loss underwent size-selection NIPS for fetal chromosomal aneuploidy, and plasma sample of pregnant women with female fetuses only underwent standard NIPS. Demographic information including maternal age, GA, and BMI were recorded. Study inclusion criteria were as follows: (1) all pregnant women underwent pre-test counseling and provided written informed consent; (2) GA < 14 weeks; (3) all samples underwent fetal chromosome analysis using single-nucleotide polymorphism (SNP) array or clinical follow-up results; and (4) available cfDNA results. The Reproductive Medicine Ethics Committee of Suzhou Municipal Hospital approved this study (Approval Number: 2019004).

### cfDNA testing

Blood samples (10 ml) were collected in EDTA tubes and centrifuged at 1600 × *g* at 4 °C for 10 min and again at 16,000 ×*g* for 10 min to obtain cell-free plasma. Plasma (600 µL) was used for cfDNA extraction using TIANamp Micro DNA purification kits (Tiangen Biotech, Beijing, China). The cfDNA library was constructed using an Ion Plus fragment library kit (Thermo Fisher Scientific, Waltham, MA, USA). The libraries were then size-selected using E-Gel EX 2% gels (Invitrogen, Carlsbad, CA, USA). The size of the selected fragment was < 140 bp as previously described [12–14]. In this interval, fetal-derived fragments were retained as much as possible, whereas more maternal components were removed. The selected fragments were sequenced using the Ion Proton system, after which all data were mapped to the hg19 human reference genome using BWA software. Low-quality reads, unmapped reads and duplicate reads were removed. All chromosomes were divided into same size segments, called bins. Bins of 20 kb was used for subsequent analyses. Locally weighted scatterplot smoothing (LOESS) regression was applied to calculated GC corrected read counts, to eliminate bin counts for biases correlated with GC [15]. The Z-score for the chromosome was calculated by the following equation: Z-score for percentage chromosome of interest in test sample = [(percentage chromosome of interest in test case) − (mean percentage chromosome of interest in reference controls)]/(standard deviation of percentage of interest in reference controls) [16]. FF for pregnancies with male fetuses were evaluated by calculating the proportion of reads from chromosome Y [17] and the FF for pregnancies with female fetuses was assessed by SeqFF, which uses a multivariate model [18]. Fetal aneuploidy status for whole chromosomes was determined using Z-score (normal, − 3 < Z < 3). R (version 4.0.4) and perl (version 5.34.0) was used for the data analysis.

### SNP array analysis

The Affymetrix CytoScan platform (Affymetrix, Santa Clara, CA, USA) was used for SNP array analysis using a previously described method [19]. Genomic DNA (250 ng) was digested, ligated, PCR-amplified, purified, fragmented, labeled, and hybridized to the Affymetrix 750 K array, which included 550,000 CNV markers and 200,000 SNP markers. After washing, staining, and scanning of arrays, raw data were analyzed using Chromosome Analysis Suite (ChAS) 3.2 (Affymetrix, Santa Clara, CA, USA). CNVs were called at a minimum length of 50 kb containing at least 20 contiguous markers, and interpreted according to the standards and guidelines for interpretation and reporting of postnatal constitutional copy number variants released by the American College of Medical Genetics [20].

### Statistical analyses

Descriptive data are presented as the median and interquartile range (IQR). Univariate and multivariate linear regression analyses were used to examine the associations of fold-change of FF with GA, BMI, FF without enrichment, and average size of cfDNA. Average size of cfDNA was categorized as ≤ 120, 121–125, 126–130, 131–135, and 136–140 bp. We also computed estimates and 95% confidence intervals (CI) for the mean differences in FF for each category of cfDNA levels. We used three different models to explore the association between the different groups of average size of cfDNA (bp) and fold-change of the FF. Model 1 was a univariate linear regression of the relationship between the average size of cfDNA (bp) and fold-change of the FF. Model 2 was adjusted for BMI and FF without enrichment. Model 3 added GA to the model based on Model 2. SPSS version 26.0 (IBM Corp, Armonk, NY, USA) was used for the data analysis. All *P* values were two-sided, and statistical significance was set at *P* < 0.05.

## Results

### Sample characteristics

The pregnancy plasma sample characteristics are presented in Table 1. The results from 102 pregnancies with male fetuses showed that maternal age, BMI, GA, uniquely mapped reads, average size of cfDNA, FF with enrichment, FF without enrichment, and fold-change of the FF were 30 years (27 to 32), 21.3 (20.1 to 23.2), 8 weeks (7 to 9.3), 2.8 Mb (2.3 to 3.5), 129 bp (124 to 136), 15.7% (10.4 to 25), 6.1% (4.1 to 8.6), and 2.8 (2.1 to 3.4), respectively. In the follow-up of 208 pregnancies, 8 had early pregnancy losses with cytogenetic results in the products of conceptions and 200 pregnancies were normal.

**Table 1:** Sample characteristics of the study population (n = 208)

Characteristics	All	Male fetus	Female fetus
Sample size	208	102	106
Maternal age (year)	30 (27 to 32)	30 (27 to 32)	29 (23 to 32)
BMI (kg/m^2^)	21.4 (20.1 to 23.2)	21.3 (20.1 to 23.2)	22.0 (20.5 to 24.4)
Gestational age (week)	8 (7 to 10)	8 (7 to 9.3)	10 (8.5 to 11.5)
Uniquely mapped reads (Mb)*	2.8 (2.3 to 3.5)	2.8 (2.3 to 3.5)	2.4 (1.9 to 3.7)
Average size of cfDNA (bp)	129 (124 to 135)	129 (124 to 136)	127 (123 to 132)
Fetal fraction with enrichment (%)	NA	15.7 (10.4 to 25)	NA
Fetal fraction without enrichment (%)	NA	6.1 (4.1 to 8.6)	NA
Foldchange of fetal fraction	NA	2.8 (2.1 to 3.4)	NA

^*^The uniquely mapped reads are the result of size selection NIPS

### Performance of cfDNA testing with or without enrichment

**Fig. 1:**
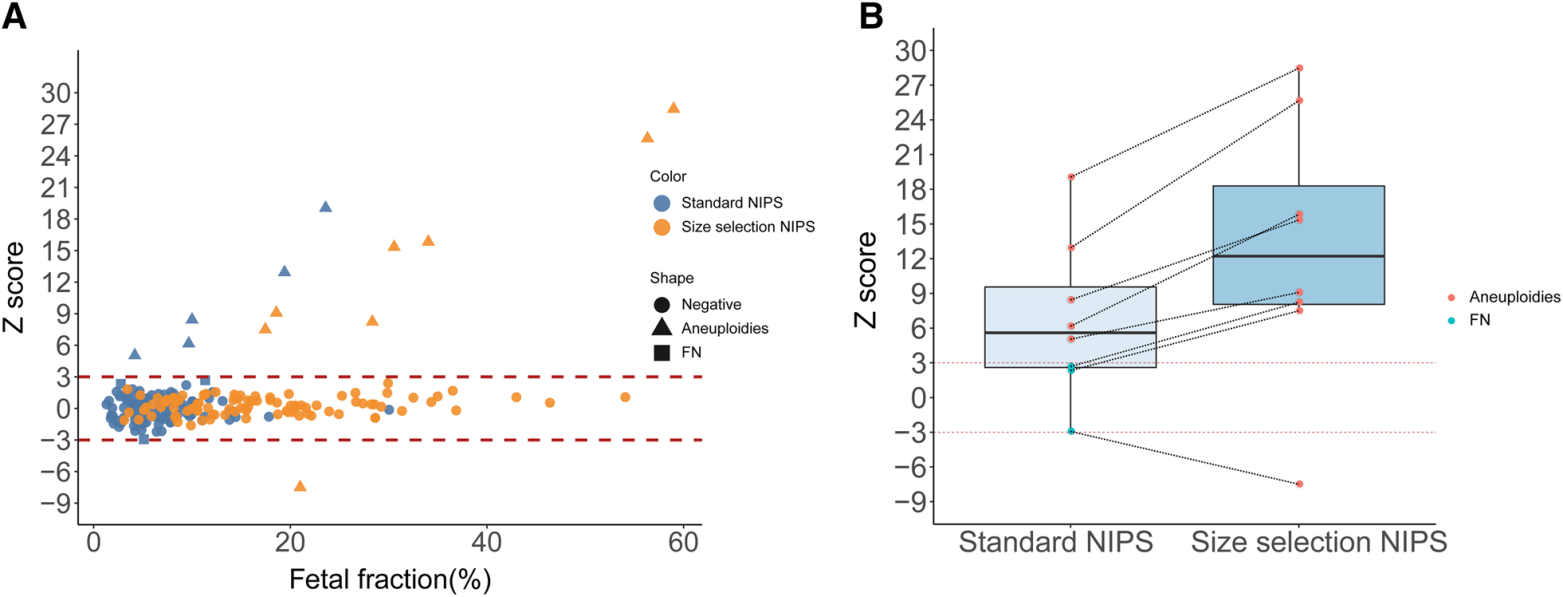
Size-selection NIPS can remarkably improve the detection of fetal chromosomal aneuploidy compared with standard NIPS. **A** Z-score according to fetal fraction between two different NIPS methods; **B** Z-score is increased in size-selection NIPS for samples with fetal chromosomal aneuploidy. Red dotted line indicates the Z-score cutoff between screen-negative and screen-positive results. FN: false negative

Z-score between − 3 and 3 indicate no aneuploidy present for each chromosome, and a higher or lower z-score suggests that trisomies or monosomies are more likely to be detected. As expected, size-selection NIPS significantly increased z-scores for every aneuploid or sample, whereas the Z-score for normal samples was unchanged, suggesting that larger Z-score separation between negative and positive samples improves the ability to distinguish such samples, thereby reducing the chance of false negatives and false positives, and increasing analytical performance NIPS. (Fig. 1A). In particular, there were three samples that were negative in standard NIPS tests. After size-selection enrichment, the Z-score of these samples was significantly improved, and positive results were obtained, which were consistent with the cytogenetic results of the fetuses (Fig. 1B). If FF was used without a threshold of 4%, the sensitivity of standard NIPS tests in detecting aneuploidy was 62.5% (5/8), and the size-selection NIPS significantly increased the sensitivity to 100% (8/8; Table 2). Specifically, case 5, 6 and 7 did not detect aneuploidy by standard NIPS tests, while the Z-score was significantly improved after size-selection NIPS, and the test results were positive (Table 2; Additional file 1: Fig. S1). Moreover, there were no false positive results for the 208 samples detected using size-selection NIPS and 169 samples detected using standard NIPS due to without remaining samples.

**Table 2:** Performance of cfDNA testing with or without enrichment for detecting fetal chromosomal aneuploidy in early pregnancy loss

	SNP array result	cfDNA testing without enrichment		cfDNA testing with enrichment	
		FF (%)	Z-score	FF (%)	Z-score
Case1	47, XY, + 21	23.6	19.0	59.0	28.5
Case2	47, XY, + 21	19.4	12.9	56.3	25.7
Case3	47, XY, + 22	9.7	6.2	34	15.8
Case4	47, XY, + 18	4.2	5	18.6	9.1
Case5	47, XX, + 8	11.3	2.7	28.3	8.2
Case6	47, XY, + 22	2.8	2.3	17.5	7.5
Case7	45, XO	5.1	− 2.9	21	− 7.5
Case8	47, XY, + 13	10	8.4	30.1	15.3

### Multiple factors affecting fold-change of the FF

**Fig. 2:**
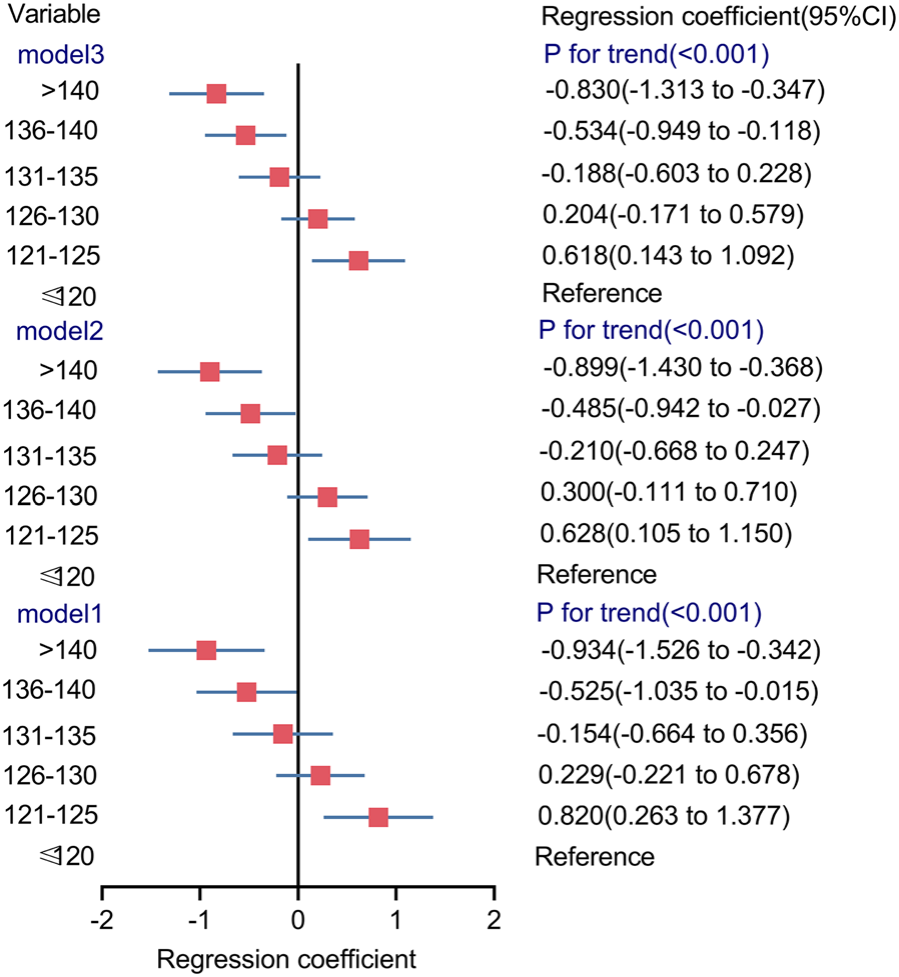
Association between different groups of average size of cfDNA (bp) and fold-change of FF

We also examined the factors that affected fold-change of the FF resulting from size-selection enrichment. Fold-change of the FF was negatively correlated with cfDNA size in both univariate and multivariate linear regression analyses (multivariate estimate − 0.035 per 1 bp, *P* < 0.0001, Table 3). Notably, when cfDNA sizes were less than 125 bp with size-selection enrichment, the mean fold-change of the FF was significantly higher than 3.23-fold (Fig. 2), suggesting that sequencing of shorter cfDNA fragments (< 125 bp) significantly increased the fold-change, thus reducing the probability of test failures at the earliest GA (6–8 weeks). We also found that fold-change in the FF was positively correlated with BMI and GA. Conversely, the FF without enrichment decreased with increased fold-change of the FF.

**Table 3:** Regression analysis of factors for predicting fold-change of fetal fraction in 102 pregnancies with male fetuses

Independent variable	Univariate analysis		Multivariate analysis	
	Regression coefficient (95%CI)	*P*	Regression coefficient (95%CI)	*P*
Gestational age (week)	0.151 (0.070–0.232)	< 0.001	0.167 (0.094–0.241)	< 0.001
BMI (kg/m^2^)	0.120 (0.071–0.170)	< 0.001	0.075 (0.031–0.119)	< 0.001
Fetal fraction without enrichment (%)	− 0.040 (− 0.079 to − 0.002)	0.040	− 0.070 (− 0.103 to − 0.037)	< 0.001
Average size of cfDNA (bp)	− 0.043 (− 0.063 to − 0.023)	< 0.001	− 0.035 (− 0.051 to − 0.018)	< 0.001

### The relationship between cfDNA fragments and fold-change of the FF

The unadjusted and adjusted associations between cfDNA fragment length categories and fold-change of the FF are presented in Fig. 2. The adjusted mean differences in fold-change of the FF across the cfDNA fragment length categories were 0.618 (95% CI 0.143 to 1.092) for 121–125 bp, 0.204 (95% CI − 0.171 to 0.579) for 126–130 bp, − 0.188 (95% CI, − 0.603 to 0.228) for 131–135 bp, − 0.534 (95% CI, − 0.949 to 0.118) for 136–140 bp, and − 0.830 (95% CI, − 1.313 to 0.347) for > 140 bp as compared to sizes ≤ 120 bp (*P*_*trend*_ < 0.0001), suggesting sequencing of shorter cfDNA fragments (< 125 bp) significantly increased the fold-change, whereas sequencing of longer cfDNA fragments (> 135 bp) significantly decreased fold-change (Fig. 2).

### Recommendation of size-selection NIPS after 7 weeks of GA

**Fig. 3:**
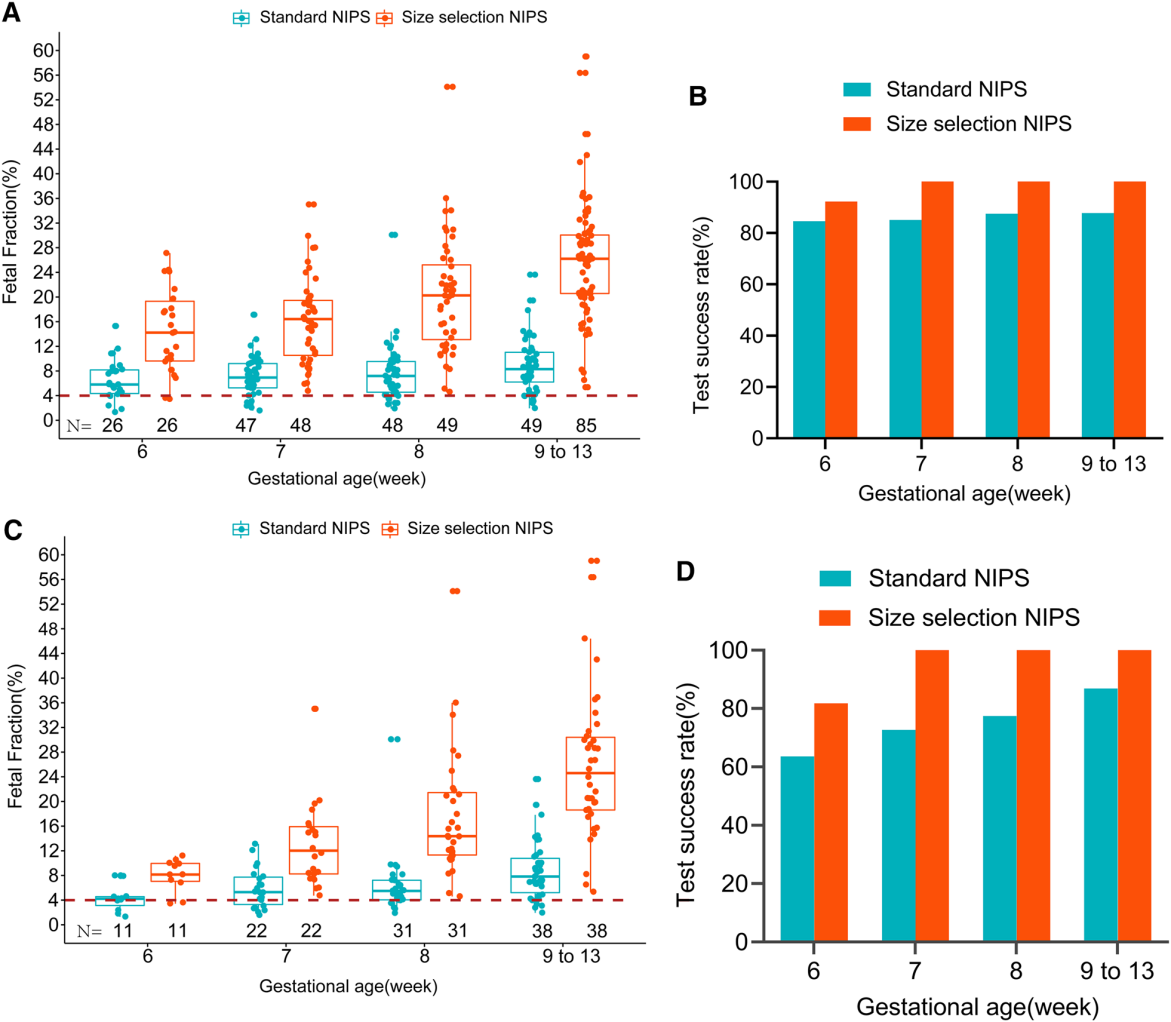
FF and test failures rate association with GA between two different NIPS methods. **A** Compared to the standard NIPS test, mean FF was increased across different GAs in total samples (6.5% vs 14.6%, 9.4% vs 16.4%, 8.9% vs 20.4%, and 10.2% vs 26.2%). **B** Compared to the standard NIPS test, test success rate was increased across different GAs in total samples (84.6% vs 92.3%, 85.1% vs 100%, 87.5% vs 100%, and 87.8% vs 100%). **C** Compared to the standard NIPS test, mean FF was increased across different GAs in pregnant women with male fetuses using size-selection NIPS (4.3% vs 8.1%, 5.9% vs 13.0%, 6.3% vs 17.7%, and 8.8% vs 25.8%). **D** Compared to the standard NIPS test, test success rate was increased across different GAs in pregnant women with male fetuses using size-selection enrichment (63.6% vs 81.8%, 72.7% vs 100%, 77.4% vs 100%, and 86.8% vs 100%)

It has been observed that pregnancies with earlier GA tend to have a low FF and cause increased test failure. We then classified GA into the following categories: 6, 7, 8, and > 9 weeks. The number of pregnant women with GA in these categories was 26, 48, 49, and 85, respectively (Fig. 3A). Compared to the standard NIPS test, the mean FF and test success rate was remarkably increased with size-selection NIPS test: (Fig. 3A, B). We further investigated the effect of size-selection enrichment on the pregnant women carrying male fetuses. The number of pregnant women carrying male fetuses with GA in these categories was 11, 22, 31, and 38, respectively. The mean FF, test success rate, and fold-change of the FF were higher with size-selection enrichment: the mean FF across GA categories was 8.1%, 13.0%, 17.7%, and 25.8% (Fig. 3C); success rates across GA categories were 81.8%, 100%, 100%, and 100% (Fig. 3D); fold-change of the FF across GA categories was 2.1, 2.4, 3.0, and 3.2-fold, respectively. The mean FF and test success rate were lower in standard NIPS tests: the mean FF across GA categories was 4.3%, 5.9%, 6.3%, and 8.8%, respectively (Fig. 3C), and the success rates across GA categories were 63.6%, 72.7%, 77.4%, and 86.8%, respectively (Fig. 3D). Together, the FF enrichment method is a reasonable strategy to detect fetal chromosomal aneuploidy in early pregnancy loss with a higher test success rate after 7 weeks of GA.

## Discussion

Here, we validated the performance of cfDNA testing that applies the size-selection enrichment technology to every sample at the earliest GA (6–13 weeks). The findings of higher test success rate and sensitivity using the FF enrichment method in earlier GA (> 7 weeks) have implications for size-selection NIPS and is highly reliable when applied to samples collected from pregnancies, including early pregnancy loss, in the first trimester after 7 weeks. To the best of our knowledge, a similar evaluation of the characteristics of cfDNA testing with and without enrichment at the earliest GA has not been published to date.

In our proof-of-concept research, we demonstrated that the sensitivity of the standard NIPS tests for detecting aneuploidy was 62.5% (5/8) and that the size-selection NIPS significantly increased the sensitivity to 100% (8/8). The overall performance of standard NIPS in early pregnancy loss, particularly for high-risk of fetal trisomies, was consistent with the sensitivity reported in three recent prospective NIPS studies [5, 21, 22]. In comparison, Colley et al. [21] identified 59% of chromosomal abnormalities in which there were available products of conception tissue cytogenetic results; Yaron et al*.* [5] found that the sensitivity of cfDNA in detecting aneuploidy in pregnancy loss was 55% (30/55), while the sensitivity could be increased to 82% (45/55) using pregnancy loss-specific LLR thresholds; Clark-Ganheart et al*.* [22] showed 66.6% (4/6) sensitivity of cfDNA testing. Together, these results suggest that standard NIPS detection of chromosomal abnormalities at an earlier GA has limited performance. We and others [10–12] developed a new method to enrich shorter cfDNA fragments (< 140 bp) to improve the FF (2.3-fold) and analytical performance of NIPS in the second trimester, suggesting that this new method may be implemented at an earlier GA. It was shown that median FF was 7.09%, which was very close to the threshold of 4%, and the FF was negatively correlated with the gestational age. Detection of fetal chromosomal aneuploidy at an earlier gestational age (6 and 7 weeks) may result in more detection failures, so the choice of enrichment method is particularly important. Our group developed a new cfDNA enrichment method that size selection of cfDNA fragments < 140 bp was performed after emulsion PCR during library construction using E-Gel^®^ Agarose Gel Electrophoresis System to remarkably increased the FF, which may be more efficient[12]. Indeed, we found that the FF enrichment method is a reasonable strategy to increase the performance of each type of fetal chromosomal aneuploidy in early pregnancy loss after 7 weeks of GA.

It has recently been reported that low FF (< 4%) samples with standard NIPS had a higher FF gain, with an average of 3.9-fold increase in the FF after undergoing the enrichment method [10]. Indeed, we found a negative correlation between FF without enrichment and fold-change of the FF and other factors influenced FF gain such as cfDNA size, BMI, and GA by using univariate and multivariate linear regression analyses. Notably, fold-change of the FF was positively correlated with GA, suggesting that enrichment technology limited the improvement of the FF at an earlier GA. Our subsequent analysis also established the following: the detection success rate was found to be only 81.8% at 6 weeks, and the success rate reached 100% at 7 weeks using the enrichment technology. Hence, the implementation of size-selection NIPS can achieve reliable results after 7 weeks of GA.

Size-selection NIPS has a broad range of detection of fetal chromosomal abnormalities and increases the analytical performance of standard NIPS [10]. However, there are still some chromosomal abnormalities that can be missed, including polyploidies, unbalanced structures, false positives, and false negatives. Moreover, this new method cannot replace cytogenetic testing for early pregnancy loss. Yaron et al*.* [5] revealed an interesting strategy where NIPS can serve as an alternative to cytogenetic analysis in guiding further management of early pregnancy loss; if cfDNA testing demonstrates aneuploidy, no further action is taken and if no abnormality is detected, the recommended early pregnancy loss workup is performed [5]. This strategy reduces the number of patients undergoing unnecessary workups, resulting in overall cost savings [8].

Knowing the genetic result of miscarriage can be helpful in counseling patients about the prognosis of future pregnancies, providing psychological support, and relieving miscarriage-related guilt. Size-selection NIPS can serve as an alternative to cytogenetic analysis in guiding further management of early pregnancy loss. Further studies are required to strengthen the clinical effectiveness of this strategy so that it can be applied clinically.

## Supplementary Information

**Additional file 1:** **Figure S1.** Size-selection NIPS can remarkably decrease the false negative of standard NIPS in the case 5 and 6. (A–C) SNP array, cfDNA testing without enrichment and cfDNA testing with enrichment results in case 5. (D–F) SNP array, cfDNA testing without enrichment and cfDNA testing with enrichment results in case 6.

## Data Availability

The datasets used and/or analysed during the current study are available from the corresponding author on reasonable request.

## Abbreviations

BMI: Body mass index
cfDNA: Cell free DNA
CMA: Chromosomal microarray
CI: Confidence intervals
FF: Fetal fraction
GA: Gestational age
NIPS: Noninvasive prenatal screening
SNP: Single-nucleotide polymorphism
