# Supplementary material for: A fetal fraction enrichment method reduces false negatives and increases test success rate of fetal chromosome aneuploidy detection in early pregnancy loss
Source: J Transl Med. 2022 Aug 2;20:345. doi: 10.1186/s12967-022-03555-9 (PMC9344718; doi:10.1186/s12967-022-03555-9)

Figure S1. **Size-selection NIPS can remarkably decrease the false negative of standard NIPS in the case 5 and 6. (A-C)** SNP array, cfDNA testing without enrichment and cfDNA testing with enrichment results in case 5. **(D-F)** SNP array, cfDNA testing without enrichment and cfDNA testing with enrichment results in case 6.


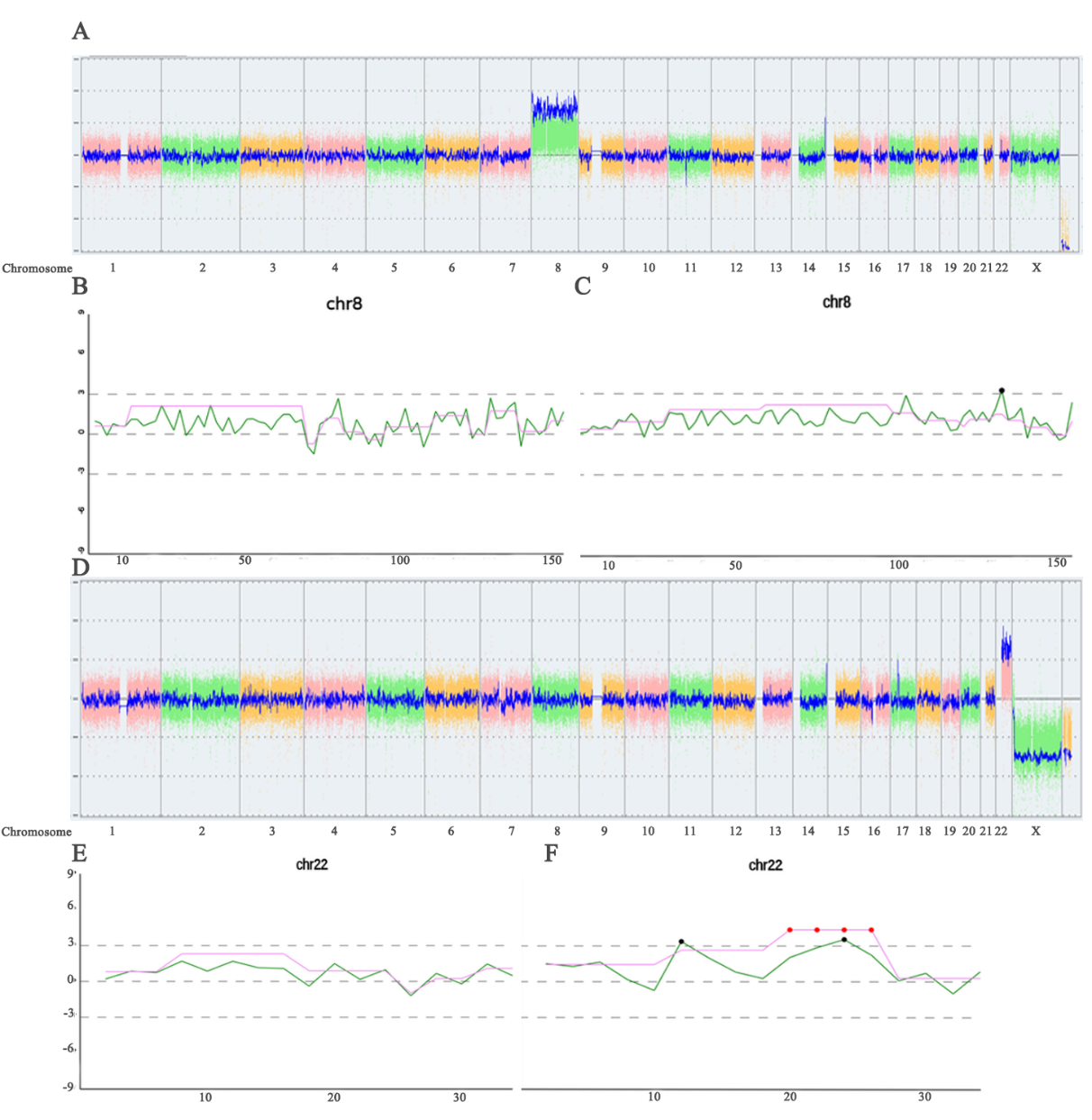

Supplement: Supplementary file 1 — Additional file 1: Figure S1. Size-selection NIPS can remarkably decrease the false negative of standard NIPS in the case 5 and 6. (A–C) SNP array, cfDNA testing without enrichment and cfDNA testing with enrichment results in case 5. (D–F) SNP array, cfDNA testing without enrichment and cfDNA testing with enrichment results in case 6. [file 12967_2022_3555_MOESM1_ESM.docx]
